# Dual‐Laser Laser‐Induced Liquid Bead Ion Desorption Mass Spectrometry: A New Approach for Adjustable Softness and Higher Sensitivity

**DOI:** 10.1002/cphc.70492

**Published:** 2026-07-16

**Authors:** Niko Popovic, Jonathan Schulte, Anne Mayer, Clemens Glaubitz, Nina Morgner

**Affiliations:** ^1^ Institute of Physical and Theoretical Chemistry Goethe‐University Frankfurt/Main Germany; ^2^ Institute for Biophysical Chemistry and Center for Biomolecular Magnetic Resonance (BMRZ) Goethe University Frankfurt am Main Germany

**Keywords:** ion yield, LILBID‐MS, native mass spectrometry, soft ionization

## Abstract

Biomechanical investigation of native protein complexes is essential to understand structural features, assembly pathways, and dynamic interactions. As sample complexity increases, current analytical platforms reach performance limits, highlighting the need for improved instrumentation. LILBID‐MS has proven to be a highly suitable native mass spectrometry method for identifying affinities, complexes, stoichiometries, and assembly pathways, even for challenging membrane proteins. In LILBID‐MS, microdroplets containing the sample are irradiated by a mid‐IR laser pulse, leading to the desorption of the sample into the gas phase without additional active charging, preserving native‐like interactions. Ion yield and in‐source dissociation both depend on laser energy, creating a trade‐off between signal intensity and soft conditions. Increasing laser energy results in higher ion counts but also boosts in‐source dissociation; consequently, weak protein interactions are difficult to detect. Here, we present a dual‐laser LILBID‐MS approach, which employs two laser pulses with separately controllable intensities and time delay. This new setup allows optimizing the degree of in‐source dissociation independently of ion count. This increased control provides the option to adapt to sample‐specific requirements, e.g., allowing for soft condition measurements, which were previously inaccessible.

## Graphical abstract

Native protein complexes often require gentle ionization to preserve weak interactions, yet higher ion yield typically goes hand in hand with increased in‐source dissociation. Our dual‐laser LILBID approach decouples ion production from dissociation by using two independently tunable pulses, enabling sample‐specific soft conditions and expanding access to previously inaccessible assemblies.

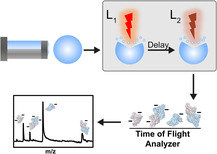

## 1 Introduction

Noncovalent interactions are a fundamentally important subject for understanding cellular processes, as a large majority of mechanisms are mediated by noncovalent interactions. Prominent examples include signal transduction, the assembly of large molecular machines, and molecular recognition events [1, 2]. Investigations into mechanisms that depend on noncovalent interactions are therefore indispensable for unraveling cellular functions [1, 3]. Native mass spectrometry (MS) has emerged as a reliable technique with regard to analyzing noncovalent interactions, as protein complexes are observed under near physiological conditions, thereby keeping protein complexes intact, including their stoichiometries, affinities, and assembly pathways [3].

Among native MS methods, laser‐induced liquid bead ion desorption mass spectrometry (LILBID‐MS)MS has proven to be an excellent method for observing fragile noncovalent interactions while being comparably tolerant toward a wide range of buffers or, if required, membrane mimics [4, 6].

LILBID‐MS soft ionization is achieved by introducing microdroplets of the aqueous sample via a droplet generator into the mass spectrometer [4, 5]. In the mass spectrometer, each microdroplet is irradiated by a mid‐IR laser pulse, which is absorbed by the water molecules surrounding the analyte. This creates an explosive expansion of the droplet, leading to the desorption of the sample into the gas phase without actively charging the protein [4]. At low laser energies, this gentle desorption mechanism allows for the preservation of most noncovalent interactions, making LILBID‐MS a useful method for investigating biomolecular complexes [4, 7].

However, a major limitation of LILBID‐MS is the intrinsic correlation between desorption laser energy and both ion yield and in‐source dissociation [5, 7]. An increase in laser intensity leads to more energy being transferred from the laser to the droplet, which, in turn, causes a higher desorption efficiency. Simultaneously, the consequential rise in energy density results in more complex dissociation, creating a trade‐off between sensitivity and softness [7, 8].

One contributing factor to a low ion yield in the LILBID process is the limited portion of the sample droplet that receives sufficient laser irradiation, particularly under low laser intensity conditions. While the sample droplets generally have a diameter of 30–70 micrometers, most of the laser energy is absorbed in the first few micrometers. The lower the laser energy, the smaller the penetration depth, and thereby the irradiated volume which evaporates explosively. This explosion disrupts the rest of the droplet, releasing some analyte molecules while simultaneously producing numerous smaller secondary droplets in which the analyte remains trapped. As these analyte molecules never reach the detector, this limits the desorption efficiency [9]. Higher laser energies overcome this problem to a certain degree at the cost of disrupting weak noncovalent bonds and promoting in‐source dissociation [5,  7]. This challenge is particularly troublesome for membrane proteins, which are often embedded in lipid micelles, nanodiscs, or detergent aggregates, and therefore require substantially higher energies to be efficiently released [5,  6,  10]. Consequently, the control over energy deposition and the ability to fine‐tune the balance between soft and harsh conditions remains limited [5, 8].

To address this limitation, we developed a dual‐laser LILBID approach that employs two separate laser pulses with adjustable delays and energies, thereby providing more control over the measurements. The underlying hypothesis was that a second laser pulse, delivered after a short delay, reirradiates the daughter droplets produced by the initial explosion. This would expand the effective IR‐irradiated volume and thereby increase the yield of desorbed ions. Due to this increased ion yield, the two‐step approach could allow for lower energies per pulse, enabling a softer transfer of ions into the gas phase. The potential delay between the two lasers is limited by the transit time required for the secondary droplets generated by the first pulse to leave the laser focus. At 150–300 m/s secondary droplet speed through a focal diameter of 1 mm, the transit time is estimated to be in the low microsecond range [8].

In this study, we demonstrate that dual‐laser LILBID substantially enhances method sensitivity, as evidenced by a dilution series of bovine serum albumin (BSA). Furthermore, the approach enables precise tuning between soft and harsh desorption conditions without compromising ion yield, as demonstrated by the preservation of the tetrameric concanavalin A under soft conditions and the shift of the dominant signal to the monomeric species under harsher conditions. In addition, we show that this method also facilitates the analysis of stability differences in the membrane protein Xenorhodopsin and mutant variants, which were previously inaccessible using the conventional single‐laser approach.

## 2 Results and Discussion

### 2.1 Experimental Setup

**FIGURE 1:**
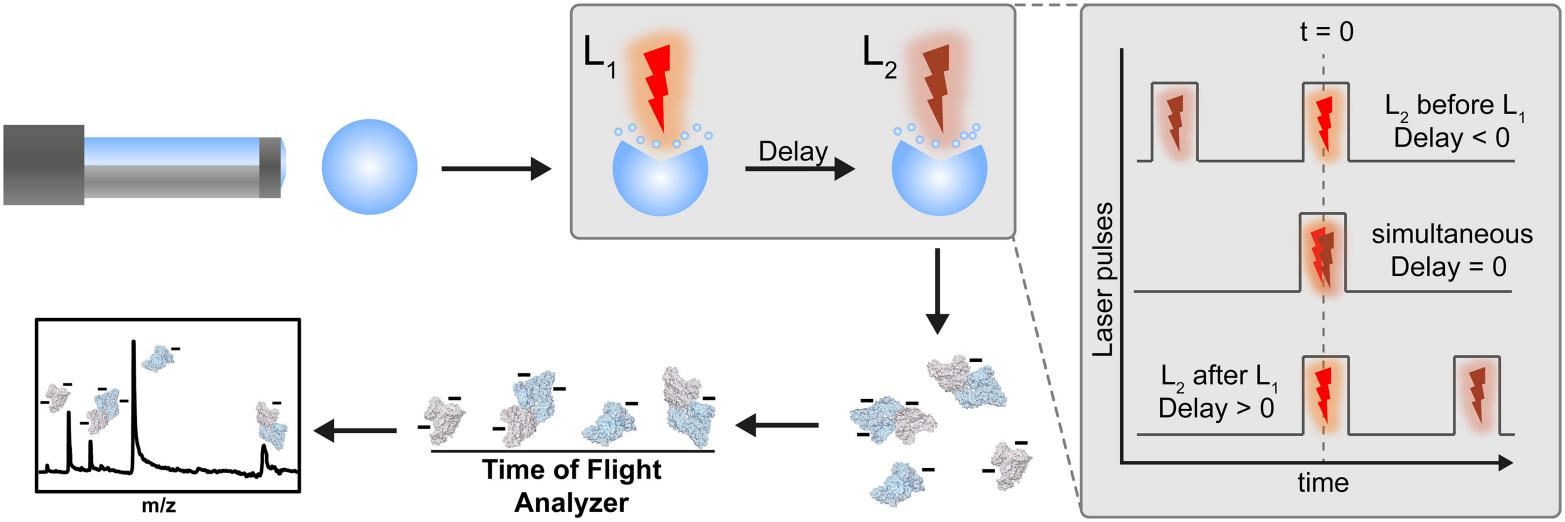
Depiction of the LILBID ionization process with a dual‐laser setup. A droplet generator emits microdroplets containing the sample of interest. These droplets are irradiated by two consecutive IR laser pulses with tunable delay, which leads to the desorption of sample ions (BSA, PCB: 3V03 [11]). These are then accelerated into a TOF analyzer and separated by their mass‐to‐charge ratio (*m*/*z*) before detection. The arrival time is calculated into an *m*/*z* range and plotted against the detector signal as mass spectrum. **Inset**: The delay between the two irradiation lasers (*L*_1_ and *L*_2_) can be chosen between −0.2 and 0.2 µs. Due to differences in beam and focal volume size, the order of irradiation is relevant for ongoing processes. Therefore, one laser (*L*_1_, bright red) is set at the fixed timepoint *t* = 0 and the second lasers (*L*_2_, dark red) timing is varied. The timing difference between the *L*_1_ and *L*_2_ pulse is given as delay. Negative delays describe an irradiation by *L*_2_ prior to *L*_1_ and a positive delay an irradiation of *L*_1_ prior to *L*_2_.

The dual‐laser LILBID configuration incorporates an additional mid‐infrared laser (*L*_2_), which is introduced into the setup in a geometry parallel to the preexisting laser (*L*_1_). It can be triggered with an adjustable delay relative to the first laser pulse (*L*_1_) (see Figure 1). Energies at which the lasers operate can be adjusted independently, thereby introducing additional degrees of freedom for precise control of the desorption process. As the values of these three parameters are central to this study, we will denote them in the following manner: $L_{1}(E_{1})\;\text{Delay}\;L_{2}(E_{2})$, where *E* *_1_* and *E*_2_ represent the pulse energies of *L*_1_ and *L*_2_, respectively, expressed in mJ, and Delay denotes the temporal separation between the two pulses, expressed in µs. Due to slight differences in the focal volume of the lasers (≈15% larger focal volume of *L*_2_), the sequence of irradiation induces differences in the resulting processes. To systematically account for the irradiation order, we define a negative delay as the condition in which *L*_2_ is triggered prior to *L*_1_, and a positive delay as the condition in which *L*_1_ is activated before *L*_2_. The aim of this study was to assess how variations in delay times and laser energies affect the sensitivity, softness versus harshness, and overall applicability of our method.

### 2.2 Detection Limit

A central question of this study was whether the dual‐laser approach is able to enhance the desorption efficiency and whether we can thereby improve the detection limit. The underlying hypothesis is that consecutive irradiation first breaks larger droplets into smaller ones, effectively increasing the available surface area for desorption during the second pulse. This, in turn, should enhance the ion yield and lower the detection threshold.

As a model system, BSA was chosen. BSA is a globular protein with a molecular weight of about 66 kDa, isolated from cow blood, and commonly used as a standard protein in biochemical research [12, 13]. Its high solubility, stability, and commercial availability make it particularly well suited as a test substance [14].

To assess the detection limit, we tested different BSA concentrations to determine the concentration limits with the single laser as well as with the dual‐laser approach. Preliminary results indicated that laser delays (LDs) longer than 0.3 µs lead to poor ionization, resulting in a low signal‐to‐noise ratio (S/N). Interestingly, this time is significantly shorter than the time after which the secondary droplets have the largest distribution in the laser focus. This indicates that additional short‐lived effects, like a transient vapor phase and remaining energy in the secondary droplets, likely support the improved ionization.

Laser energies below 4 mJ per pulse were not used, as no signal was detected beneath this threshold, and laser energies of around 10 mJ per pulse are the maximum achievable energies with our current laser setups. As softness is not relevant here, the 4 mJ settings were not of interest, and we carried out the experiments to determine the effect of the second laser on the detection limit at higher laser energies for the dual‐laser measurements with delays between −0.2 and 0.2 µs.

#### 2.2.1 Results Detection Limit

**FIGURE 2:**
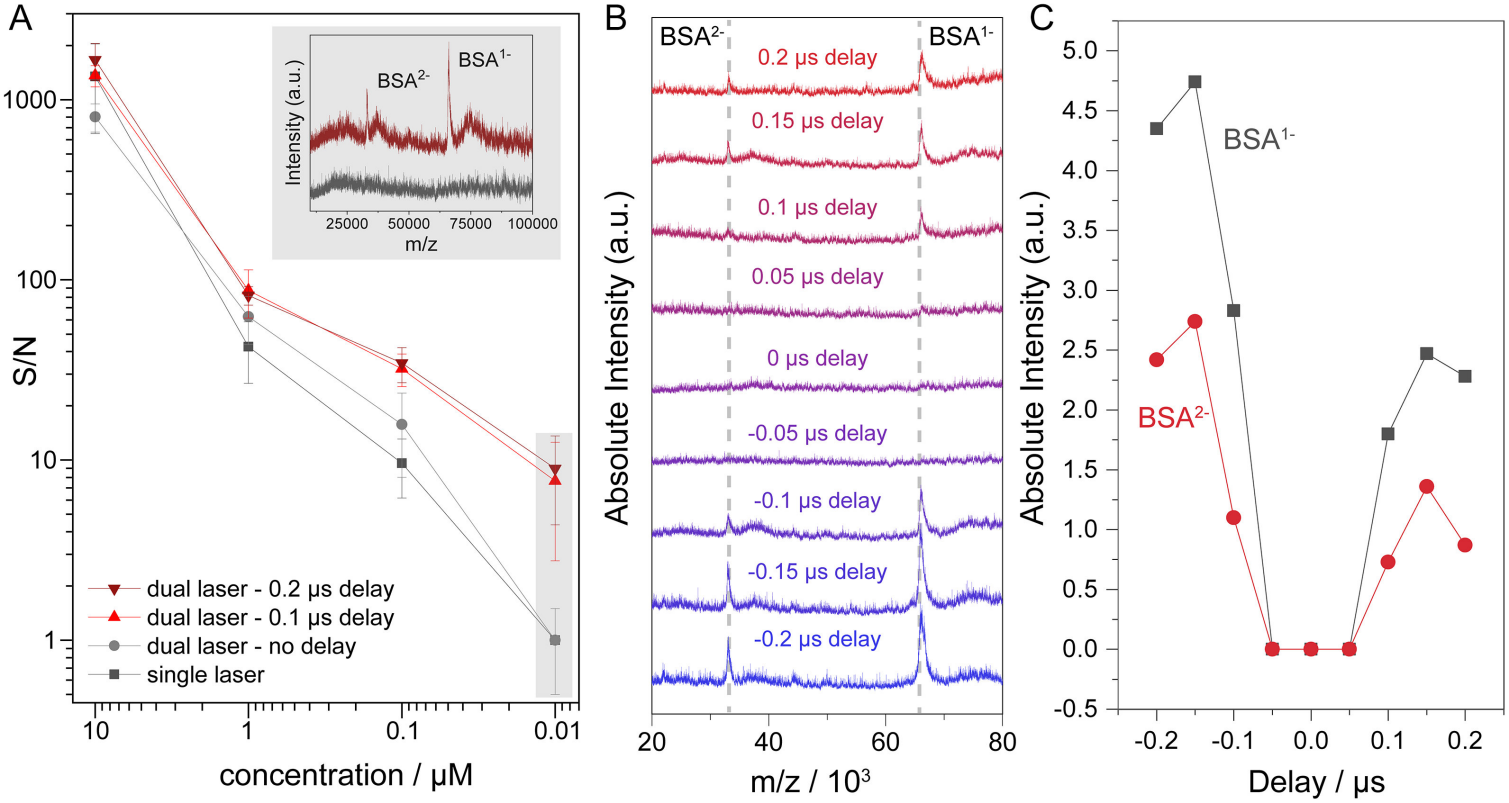
The dual‐laser approach increases the sensitivity of LILBID‐MS by nearly a magnitude. (A) The S/N of the singly charged BSA peak in LILBID‐MS spectra was plotted against the BSA concentration. The measurement points on dark gray resulted from a single laser with $L_{1}(10)$. All other plot lines were measured with $L_{1}(7)\;(0.0\;to\;0.2)\;L_{2}(7)$ (Delay 0 µs: light gray, Delay 0.1 µs: light red, Delay 0.2 µs: dark red). At 10 nM, only the dual‐laser approach with delay yielded any detectable BSA signal (inset, dark red: Delay 0.2 µs and light gray: Delay 0 µs). All points are the mean of three measurements and the error bars represent the standard deviation. (B) Spectra of 10 nM BSA and a dual‐laser approach with $L_{1}(10)\;(-0.2\;to\;0.2)\;L_{2}(10)$. The Intensities are absolute intensities in arbitrary units. The peaks of the singly and doubly charged BSA are marked with a dashed line. BSA was detectable only with delays larger than ±0.1 µs. (C) Absolute signal intensities of peaks from singly charged (gray) and doubly charged (red) BSA plotted against the LD. The highest peak intensities were achieved at a delay of −0.1 µs while delays between −0.05 and 0.05 µs resulted in no detectable BSA peaks.

The measurements revealed a clear dependence of signal intensity on both protein concentration and laser parameters. BSA could be readily detected between 10 µM and 100 nM across all tested laser energies and delays (Figure 2A). At 10 nM, however, distinct differences emerged, as no laser energy setting could be found at which either a single laser pulse or two simultaneous laser pulses could produce a mass spectrum. In contrast, sequential irradiation with delays between 0.1 and 0.2 µs and pulse energies of at least 7 mJ per laser consistently enabled reliable detection. (Figure 2B,C) This supports the notion that a two‐step irradiation sequence markedly improves ionization efficiency. At even lower concentrations (5 nM), no BSA signal could be obtained under any conditions.

Figure 2A illustrates this effect. Here, the (S/N) is shown for four setups (single laser at 10 mJ, two lasers simultaneously at 2 × 7 mJ, and two lasers with delays of 0.1 or 0.2 µs at the same total energy) over the full concentration range. The benefit of sequential pulses is particularly striking at 10 nM, where only the delayed dual‐laser setup produced analyzable signals. This is highlighted in the inset shown in Figure 2A. Notably, the dual‐laser approach with delay yielded a higher S/N than that without delay at all concentrations. The S/N of spectra recorded at 100 nM and 10 nM BSA using dual‐laser measurements with delays of 0.1 or 0.2 µs was similar to that obtained without delay for samples at concentrations an order of magnitude higher (1 µM and 100 nM, respectively).

As the spectra at 10 nM generally show low intensity, we tested if even higher laser energies could be beneficial. We analyzed the signal intensity of BSA at different LDs with 10 mJ per pulse. (Figure 2B,C) Strikingly, there was no BSA signal detectable at delays between −0.05 and +0.05 µs and there was a maximum signal intensity at delays of ±0.15 µs. More pronounced effects at negative delays are most likely a result of differences in the focal alignment of the two lasers, and hence in energy density as well as the irradiated area.

In summary, the BSA experiments demonstrated that the dual‐laser strategy significantly extends the detection limit. While conventional measurements failed at 10 nM, time delayed laser pulses produced stable signals, confirming that droplet fragmentation by the first pulse and subsequent desorption by the second pulse significantly improves the method's sensitivity.

### 2.3 Softness

**FIGURE 3:**
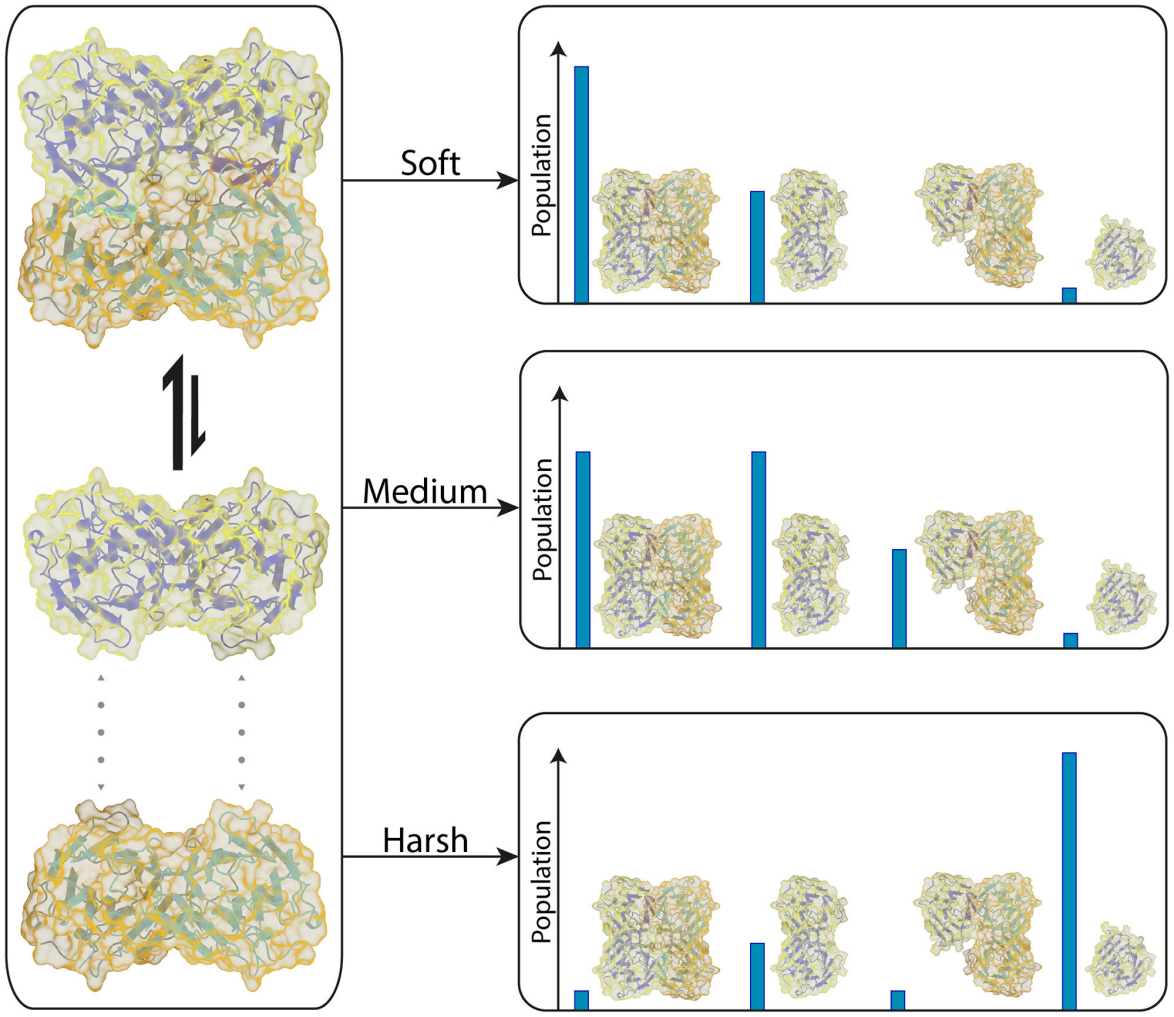
The LILBID desorption process can vary in harshness or softness, which is reflected in the oligomeric state of the desorbed species. **Left**: Concanavalin A forms a tetramer consisting of two dimers under native conditions (PCB: 1JBC [18]). At pHs larger than 5.5, the tetramer is the prevalent state, followed by the dimeric form [19, 20]. Monomer and trimer are in negligible concentrations [15, 17, 19]. **Right**: By adjusting laser parameters, the desorption process can vary significantly between soft and harsh conditions. This is reflected in the detected species (relative abundance is shown by the bars), which can either be predominantly tetrameric (top—soft conditions) or monomeric (bottom—harsh conditions) or a mixture of all possible oligomeric states between monomer and tetramer (middle—medium conditions).

The correlation of ion release and in‐source dissociation represents a key challenge in LILBID. Our hypothesis is that the dual‐laser setup can mitigate this issue by distributing the laser energy required for ion release across both time and increased droplet surface area, thereby reducing in‐source dissociation. To investigate this hypothesis, we selected concanavalin A (ConA) as a model protein. ConA is a well‐characterized protein originating from jack beans that, under physiological conditions, is coordinated in a tetrameric complex, forming a dimer of dimers. This is reflected by a strong binding interface between the first two protomers and a weaker interaction between adjacent dimers [15, 17]. Furthermore, no naturally occurring trimeric species of ConA have been reported, making ConA an excellent candidate for evaluating the softness of the dual‐laser LILBID approach, as potential trimeric signals in a mass spectrum must stem from in‐source dissociation caused by the laser irradiation [15, 17]. The exclusive observation of the naturally occurring dimeric and tetrameric species with no dissociation would be an indicator of the softness of the dual‐laser approach. In contrast, harsher conditions would be reflected by a shift toward dimeric and monomeric species, accompanied by the appearance of trimer, as the tetrameric complex undergoes dissociation (Figure 3). At sufficiently harsh conditions, the predominant mass observed should be the monomeric species. ConA was used to systematically assess whether the dual‐laser approach is able to control the balance between preserving intact complexes, inducing controlled dissociation, and optimizing ionization.

In order to observe conditions under which soft and harsh ionization occurs, we had to investigate different settings of laser energies and delays. For the measurements, we therefore set both lasers to one of three energy settings: 4/7/10 mJ and varied the LD between −0.2 and 0.2 µs in 0.05 µs increments. Positive delays indicate that laser *L*_1_ irradiates the droplet before laser *L*_2_, while negative delays correspond to the opposite order. This yields 81 possible parameter combinations, all of which were systematically scanned.

#### 2.3.1 Results Softness

**FIGURE 4:**
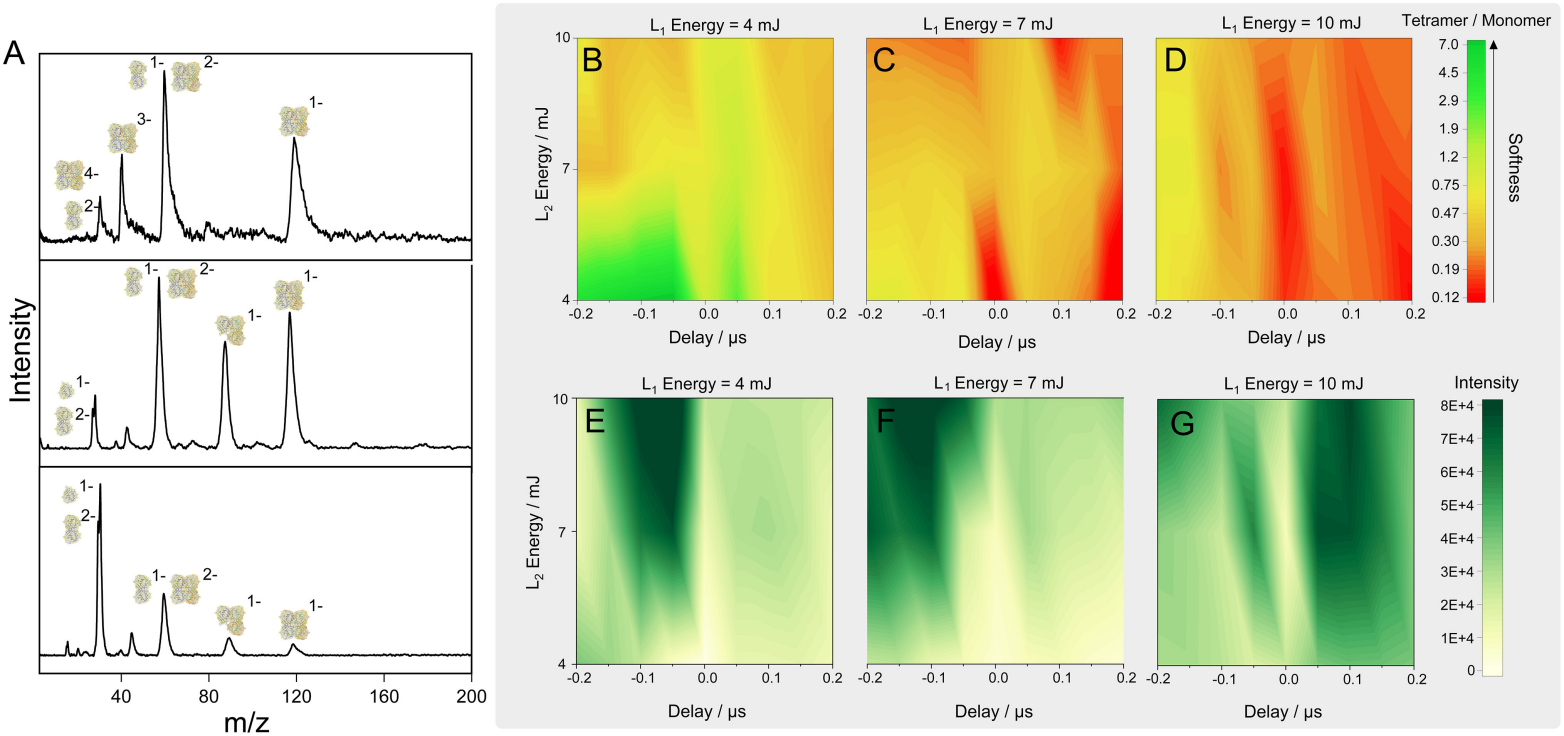
Dual‐laser measurements with delays between  −0.2 and +0.2 µs and laser energies varying between 4 mJ and 10 mJ per pulse allow for adjustments of the LILBID desorption process with regard to harshness and sensitivity. (A) Spectra of concanavalin A (PCB: 1JBC [18]) measured with soft (top), medium (middle), and harsh (bottom) conditions. The top spectrum shows mainly tetramer with little dimeric contribution, the spectrum in the middle contains all oligomeric species from monomer to tetramer, and the bottom spectrum is dominated by the monomer peak. (B–D) The heatmaps indicate harshness of the settings and show ratio of the peak integrals for the singly charged tetrameric species (*m*/*z* $\approx 116\cdot 10^{3}$) and monomeric species (*m*/*z* $\approx 29\cdot 10^{3}$) in dependence of the *L*_2_ energy and the LD. *L*_1_ energies are 4 mJ (B), 7 mJ (C), and 10 mJ (D). Red represents the harshest conditions (mostly monomeric) and green the softest conditions (mostly tetrameric). Simultaneous irradiation always results in the harshest conditions with the given laser energies, while really soft conditions were only achievable with LD and low laser energies (D). (E–G) These heatmaps show the sum of the measured peak integrals (*m*/*z* $\text{\textasciitilde{}}29\cdot 10^{3},\text{\textasciitilde{}}58\cdot 10^{3},\text{\textasciitilde{}}87\cdot 10^{3},\text{\textasciitilde{}}116\cdot 10^{3}$) divided by the average background signal. dependent on the *L*_2_ energy and the LD. *L*_1_ energies are 4 mJ (E), 7 mJ (F), and 10 mJ (G). Dark green represents the highest intensities and white the lowest conditions. In all heatmaps, a bright line is visible at a delay of 0 µs, representing a drop in intensity correlated simultaneous irradiation of both lasers. Higher intensities were achieved primarily at higher laser energies.

Figure 4 summarizes the experimental findings. Panel 4A shows representative spectra for the different measurement modes. The upper spectrum corresponds to measurements under soft conditions, revealing predominantly tetrameric ConA with charge states from 1‐ to 4‐, along with a small amount of dimeric ConA (charge states 1‐ to 2‐). Under these gentle experimental conditions, no trimeric species are detected, indicating that the method does not promote in‐source dissociation. The middle spectrum was acquired under intermediate conditions, under which the trimeric species becomes detectable with signal intensities comparable to those of the tetrameric and dimeric species, whereas the monomeric species is scarcely represented. The bottom spectrum corresponds to harsh conditions and, as expected, is dominated by the monomeric species. To quantitatively assess the softness of the conditions, the ratio of the integrated signal intensities of the singly charged tetramer (peak at *m*/*z* = $118\cdot 10^{3}$) and monomer (peak at *m*/*z* = $29.5\cdot 10^{3}$) was determined. This was done for all 81 laser settings as shown in the heatmaps in Figure 4B–D. Each plot includes 27 parameter settings with a fixed energy of *L*_1_, while the delay (*x*‐axis) and the energy of *L*_2_ (*y*‐axis) were altered. Figure 4B shows that the softest desorption conditions are achieved at $L_{1}(4)\;(-0.05)\;L_{2}(4)$, for which spectra were dominated by tetrameric signals. Notably, the measurements at these laser energies with no delay, hence imitating one laser at 8 mJ, resulted in no measurable signal.

Raising the laser energy of *L*_2_ to 7 mJ or 10 mJ results in harsher conditions, which can be attributed to the increased energy transfer. This subsequently leads to a higher local energy density during the desorption event, which promotes complex dissociation. As shown in Figure 4C, increasing the energy of *L*_1_ to 7 mJ facilitates the dissociation of the protein complexes. Almost all measurements with these settings $L_{1}(7)\;(-0.2\;to\;0.1)\;L_{2}\;(4-10)$ result in medium dissociation to varying degrees. For $L_{1}(7)$, neither a change in delay nor in energy of *L*_2_ leads to a noticeable change in softness or harshness. The most dominant signals in the spectra remain the tetramer and dimer, although the increased local energy density already causes a noticeable increase in the trimer. At $L_{1}(7)\;0.2\;L_{2}\;(4-10)$, the in‐source dissociation increases analogous to the measurements at $L_{1}(4)\;0.2\;L_{2}\;(4-10)$. Finally, an increase to 10 mJ energy for $L_{1}$ leads to further in‐source dissociation. In line with the observations for $L_{1}(4/7)$, a positive delay results in harsher conditions compared to a negative delay. At this energy, tetramer and trimer abundances drop significantly, while the monomer becomes the most dominant species in the spectra, as shown in Figure 4A (bottom). Figure 4E–G shares the same axes as Figure 4B–D, but plot the signal intensity rather than the tetramer/monomer ratio. In Figure 4E, it becomes evident that signal intensity is the highest for the settings $L_{1}(4)\;(-0.05\;to\;-0.1)\;L_{2}\;(7/10)$, indicating that the ion release is more efficient with negative delays and increased laser energies. This trend is also shown in Figure 4F for the $L_{1}(7)$, but interestingly is not applicable for $L_{1}(10)$, as shown in Figure 4G. At these settings, the highest intensity is achieved at $L_{1}(10)\;(0.05\;to\;0.2)\;L_{2}\;(4-10)$. Also noteworthy is the trend that a delay of 0 µs for all energies results in the harshest settings (Figure 4B–D) and much reduced signal intensity (Figure 4E–G). In summary, systematic adjustment of LD and energies is essential to direct measurements toward either soft, medium, or harsh desorption conditions, while at the same time avoiding insufficient signal intensities. Knowledge of precise response to these parameters allows deliberate experimental control over complex dissociation behavior.

### 2.4 Membrane Proteins

Up to this point, our analyses have focused on soluble proteins, which exhibit a comparatively low propensity to retain surrounding buffer molecules. In contrast, membrane proteins maintain a strong and persistent association with their solubilizing agents, such as detergents, due to the need to shield their hydrophobic transmembrane regions. This makes membrane proteins a particularly interesting test case for the dual‐laser approach, as their hydrophobic nature and tight interactions with detergents demand careful removal of associated molecules without compromising complex integrity or signal intensity—especially given their typically low expression yields [5, 21]. In this context, LILBID‐MS is a particularly valuable tool, allowing the analysis of membrane proteins from many different membrane mimics, providing direct insights into oligomeric states, intermolecular interactions, and binding partners [5, 22].

Generally, detergent‐ or lipid‐mediated solubilization strongly affects mass spectral quality, typically producing broad, heterogeneous peaks and reduced signal intensities. Meaningful characterization would therefore benefit immensely from enhanced ion yield and efficient removal of associated detergents or lipids, with conditions that can be tuned from harsh to gentle with regard to the native oligomeric state of the complex [5, 23, 24]. The potential transfer, especially of quantitative methods [7, 8, 20] from soluble to membrane complexes, will require increased control of the LILBID desorption process.

To validate the presented approach for membrane proteins, we applied LILBID‐MS with selected desorption settings to the microbial NsXeR from the nanohaloarchaeon Nanosalina. NsXeR is an inward‐directed proton pump that physiologically assembles as a trimer and is of high interest in optogenetics [25, 26]. In addition to the wild type, two mutants were examined that are presumed to play a key role in the oligomerization of the monomers into a trimer: F50Y and R100K. Based on predictions performed with PDBePISA, F50Y seems to be the most relevant residue at the oligomeric interface, while R100K also seems to contribute. Additional analysis of the X‐ray structure (6EYU) supports these assumptions [25]. Both mutants exhibit reduced yields and an increased tendency for precipitation over time, making them demanding test cases for methodological validation. Here, we want to test the feasibility of our dual‐laser LILBID to determine the influence of these mutations on oligomerization state and complex stability.

### 2.5 Results Membrane Proteins

**FIGURE 5:**
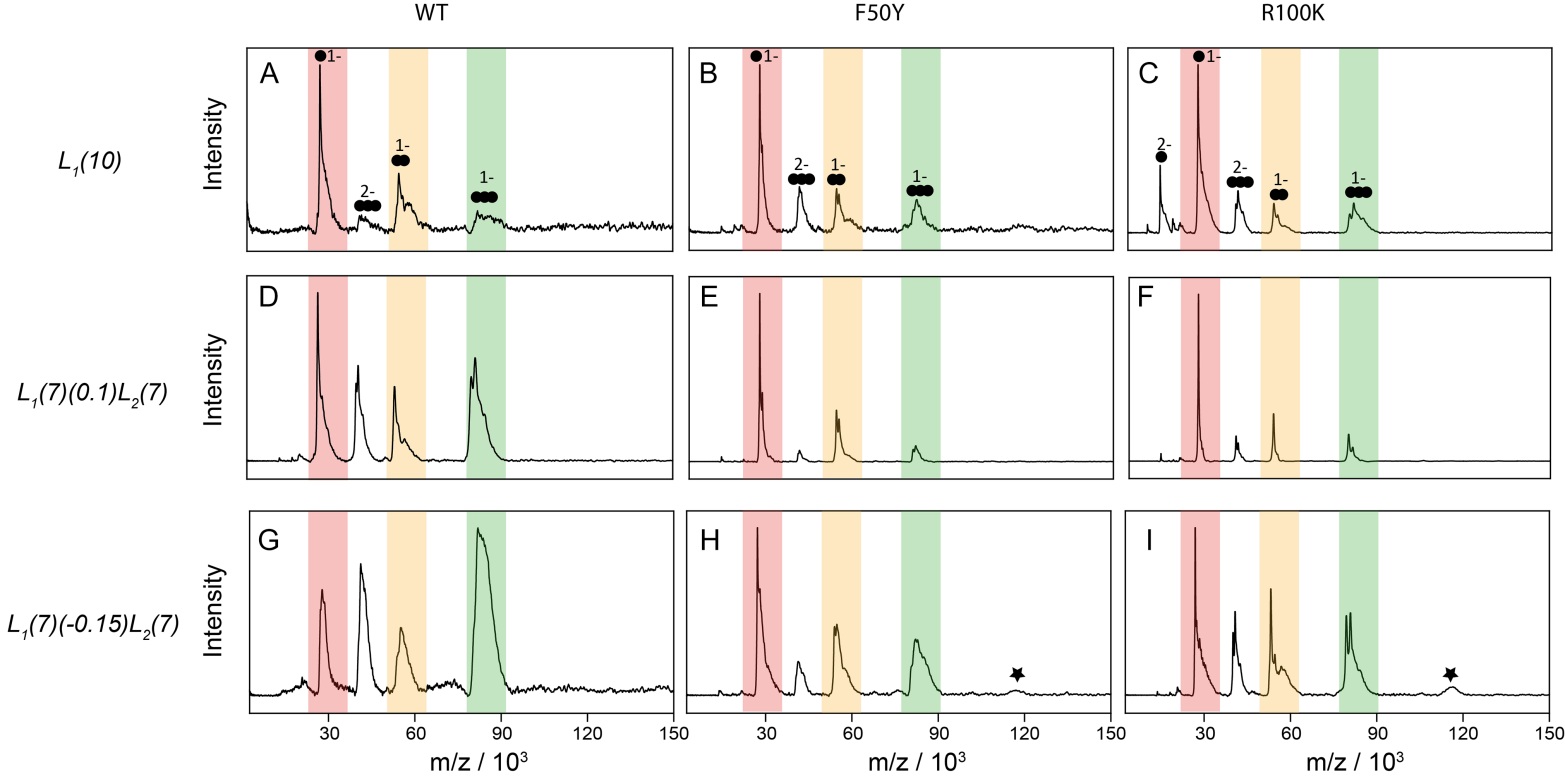
Dual‐laser approach spectra of Xenorhodopsin from Nanosalina (NsXeR) wild type and mutant variants R100K and F50Y. The colored regions (green, yellow, red) mark the peaks, which represent trimer, dimer, and monomer species, respectively. (A–C) show measurements with a single laser. (D–F) with a positive delay of 0.1 µs and (G–I) with a negative delay of −0.15 µs. Wild‐type spectra (A,D,G) show that the trimer can be preserved in abundance, especially with a negative delay here ( G) at −0.15 µs representing soft desorption conditions. Both mutant variants F50Y (B,E,H) and R100K (C,F,I) show as well more trimer for these soft conditions (H,I), albeit increased presence of dimeric and monomeric species is observed compared to the wild type (WT), across all tested delays. At these soft conditions, a tetrameric species is observed for the mutants (H,I), marked with a black star. The observed double peaks are most likely attributable to cardiolipin binding.

Our measurements revealed that all protein species could, in principle, be detected with a single laser. However, in order to achieve a sufficient S/N, to release the proteins out of the Tris DDM buffer, the use of high laser energies was required. At $L_{1}(10)$, the monomeric species appeared as the predominant signal, and the spectra revealed little differences between the WT and the mutants. If observing only these spectra, one could assume the monomer to be the naturally occurring dominating species in solution, without a clear effect of the mutants on oligomerization or complex stability. Based on our previous results, we then tested if the dual‐laser approach could be used to achieve softer measurements at comparable S/N ratios for these detergent‐solubilized membrane proteins as well. The softness study for soluble complexes (Subsection Results Softness) implicated that negative delays produce gentler ionization conditions, especially when combined with low energies per laser pulse. Starting from these settings, we explored the feasibility of using the same laser parameters or trends for our membrane complex. While the softest setting for soluble proteins was $L_{1}(4)(-0.05)L_{2}(4)$, this did not allow us to achieve reasonable mass spectra for NsXeR, as peaks were barely differentiable from noise. We attributed this effect to the detergent environment, which must be partially removed during desorption to increase ion yield, reduce ion heterogeneity, and ultimately improve spectral resolution. An increase of the laser energies to $L_{1}(7)(Delay)L_{2}(7)$ was required to observe a similar trend with regard to the delay for NsXeR. Especially negative delays resulted in soft measurements (Figure 5G–I), while positive delays were harsher in comparison (Figure 5D–F).

The softest measurements ($L_{1}(7)(-0.15)\;L_{2}(7)$) show a reduced S/N ratio, when compared to measurements with a positive delay, but they are crucial for reflecting native oligomer distributions. Under these gentle conditions, the difference between the mutant variants and the WT becomes substantially more pronounced. For the NsXeR WT, the trimer species becomes the dominant species, while the monomer and dimer species remain the dominant species for the R100K and F50Y mutants even at the softest measurement. Strikingly, F50Y and R100K displayed only low levels of trimers and an unexpected but noticeable fraction of tetrameric complexes, as shown in Figure 5H–I. This suggests that both mutations alter the oligomerization mechanism, potentially stabilizing an alternative interface or leading to unwanted aggregation. Biologically, this is intriguing since even subtle changes at the oligomeric interface may affect functionality and proton‐pumping activity.

Interestingly, all microbial rhodopsins form homooligomers (trimers in Archaea and pentamers in bacteria) [27]. In some cases, cross‐protomer interactions have been shown to play a role [28]. However, the functional relevance of the oligomeric state remains unresolved, and additional structural and biophysical data on the interface interactions are still required [29].

Therefore, investigating such mutants and their mutation‐dependent changes can provide valuable insights into native structure–function relationships and the extent to which these are preserved in the mutants.

## 3 Conclusion

In this study, we have established a dual‐laser LILBID setup extending the capabilities of native MS by introducing a novel degree of control over the LILBID desorption process. We were able to overcome the intrinsic trade‐off between ion release and soft condition measurements occurring in the single‐laser method by decoupling droplet fragmentation from ion release using two independently triggered laser pulses with an adjustable delay and laser energies. This dual‐laser LILBID setup resulted in a significant enhancement in sensitivity. The model protein BSA was used to demonstrate that the setup increases desorption efficiency up to one order of magnitude, allowing for measurements at low nanomolar concentrations, which were previously not achievable with a single‐laser setup. The setup enables consecutive laser‐energy deposition into the sample, using lower energies per pulse and an adjustable delay between pulses. This allows internal cooling and spreading of the secondary droplets, increasing the number of droplets presented to the second laser pulse and preventing excess energy transfer to the complexes of interest, which would otherwise lead to unwanted dissociation. Concanavalin A was used as a model protein to demonstrate the control over the measurements achieved by fine tuning parameters like LD and energy. This gives us the option to systematically disassemble the complex into its subunits or to keep the quaternary structure intact, while maintaining sufficient ion intensities and S/N, for sample concentrations as low as 10 nM.

At last, we demonstrated that the dual‐laser setup improves measurements even of membrane proteins significantly, both with regard to higher sensitivity and in more control over the measurements harshness. In the case of the detergent solubilized membrane protein complex NsXeR and its mutants, the dual‐laser approach showed that higher laser energies are required to achieve the same outcomes as for soluble complexes. Nevertheless, by adjusting the laser energies and the interpulse delays, it is possible to release membrane proteins from the detergent micelle while either preserving or deliberately dissociating their native oligomeric state. The possibility to define precise laser settings enables direct comparison of their effects on complex stability and thereby allows definitive conclusions about subtle influences of, e.g., mutations on the native oligomeric state—something that was not achievable with the single‐laser approach due to uncontrolled desorption‐induced dissociation or insufficient ion yields.

The heatmaps in Figure 4 show that parameter combinations yielding maximal softness or harshness (B–D) may not be optimal for the intended measurements if they simultaneously result in low signal intensities (E–G). The loss of ion intensity at harsh laser settings can be attributed to the increased kinetic energy with which ions leave the desorption point, potentially causing them to exit the region in which the Wiley–McLaren‐type ion optics, operating under delayed extraction, can efficiently accelerate them toward the detector.

**FIGURE 6:**
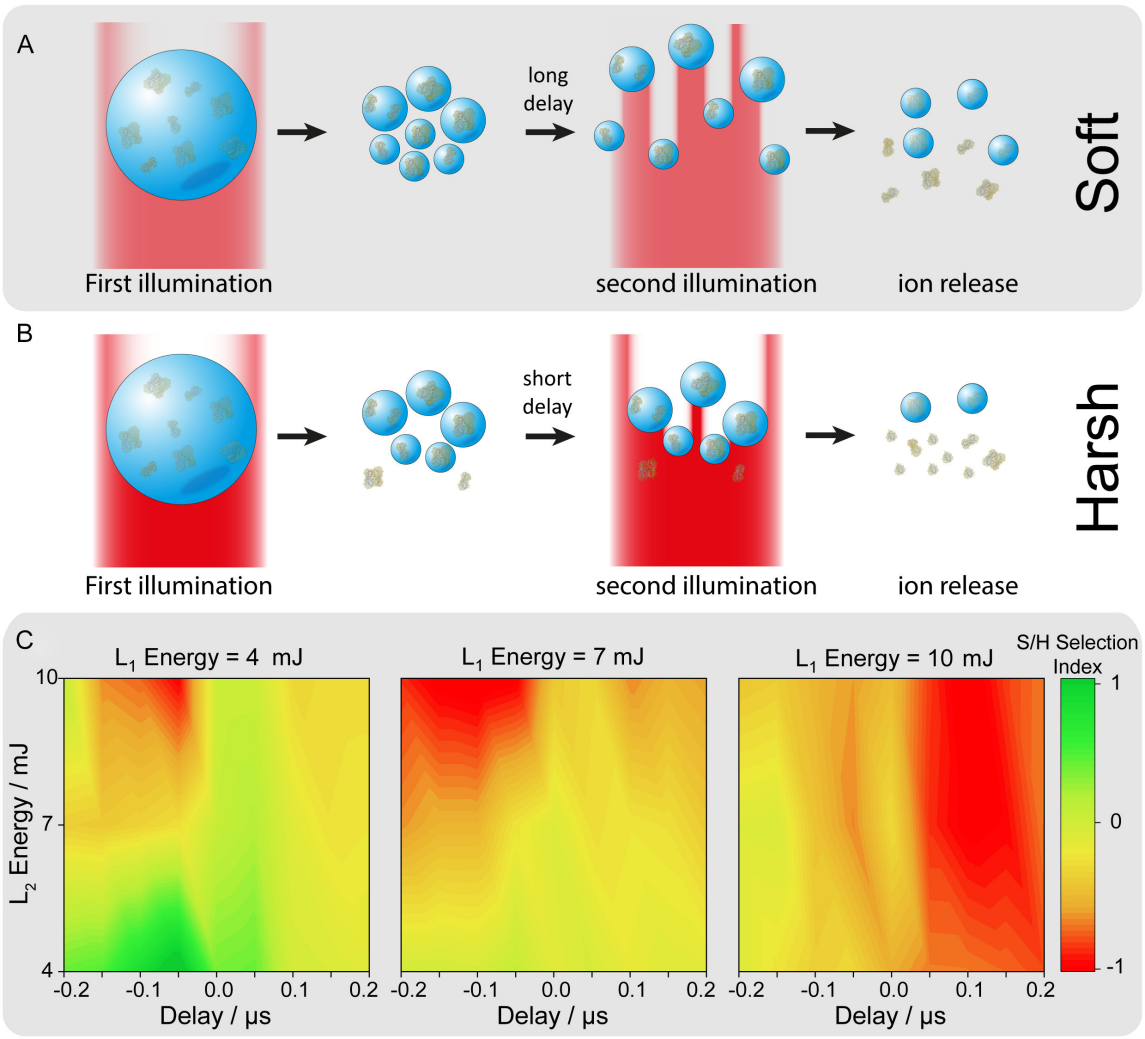
The selection of laser parameters like laser energies and delay between the laser pulses allows for the control of softness/harshness and efficiency of the desorption process. (A) First irradiation of the sample droplet with low laser energies results mainly in the droplet to burst into smaller droplets with only little energy transfer, while desorbing only very few of the sample ions. A large delay allows internal cooling and the spatial distribution of these secondary droplets, therewith bringing overall more sample volume into a position for further irradiation with the second laser pulse. With the second laser at low energy, the sample ions can be softly desorbed from the secondary droplets, while maintaining most of the noncovalent interactions. (B) By using high laser energies and a short delay, it is possible to desorb ions in both steps and to gain a high intensity. Due to the high local energy distribution, most of the noncovalent interactions are dissociated during that process. (C) Dependent on the aim of the measurement, soft or harsh conditions might be chosen, but in all cases, higher intensity is advantageous. The heatmaps show a selection index plotted against the laser parameters (laser energies, delay). Settings which should be chosen, if a high degree of desorption is advantageous (harsh measurements at high intensities), are plotted in red, while green shows that those settings, which allow to keep complexes intact (soft measurements at high intensities), are plotted in green. Yellow regions show low intensities or neither harsh nor soft conditions.

Therefore, both aspects have to be taken into account, as shown in the final heatmap of Figure 6, which integrates both resulting features and plots a selection index against the laser energies and delays. The selection index is determined by differentiating between soft and harsh conditions. The threshold is set at a tetramer to monomer ratio of 1. Larger ratios indicate rather soft and lower indicate harsh conditions. The index is then calculated by the multiplication of the signal intensities (as shown in Figure 4E–G) with either the peak ratio of tetramer to monomer for the soft conditions or with the inverse value (monomer/tetramer ratio) for the harsh conditions. Harsh conditions are indicated by a negative sign. All values are then normed between 1 and −1. Therefore, green (positive values) represents a good combination of high intensity and soft conditions and red (negative values) of high intensity and harsh conditions. Yellow can either be measurements with low intensity or with medium conditions. These maps can be used to determine the best parameters for the scope of the respective measurement. This work introduces a versatile methodological advancement that provides a previously unattainable level of control in LILBID‐MS. The improved approach is applicable to both soluble proteins and membrane protein complexes in a similar manner, although the experimental settings must be adjusted accordingly.

The general importance of membrane proteins stands in sharp contrast to the still limited analytical tools available [30, 31]. The dual‐laser strategy presented here directly addresses several of the most common challenges: poor stability, low concentrations, and weak signal quality [5, 23, 24]. By combining enhanced ion yield with tunable “softness,” the method enables reliable characterization of even complex membrane proteins and can resolve subtle differences such as the impact of a mutation on stability. Furthermore, the reduced sample requirements open the door to mutant screening or drug‐binding studies on a scale that was previously unfeasible.

A key advantage of the approach is that it delivers reliable results even with limited protein availability and reduced stability. This represents a major improvement over established methods such as blue native polyacrylamide gel electrophoresis, which suffers from dissociation effects during solubilization and dye binding, and often produces distorted mass estimates due to detergent association. The method presented, not only advances the study of rhodopsins but can also extend to pharmacologically highly relevant targets such as G protein‐coupled receptors, which are among the most challenging protein classes due to low expression levels and high instability. For such difficult systems, methodological advances like those presented here could be decisive in making structural and interaction data systematically accessible [32, 33]. Furthermore, the reduced sample requirements open the door to mutant screening or drug‐binding studies on a scale that was previously unfeasible. Future developments will include a means to quantify laser energy transfer for the dual‐laser approach to broaden our quantitative LILBID approach [7, 8, 20] for the top‐down determination of dissociation constants. Another aspect will be the determination of the transferability of the here presented results to other membrane mimics beside detergent micelles. In summary, the dual‐laser approach further strengthens and improves the capabilities of LILBID‐MS for structural, mechanistic, and functional studies of complex biomolecular assemblies.

## 4 Experimental Section

### 4.1 Chemicals and Reagents

Ammonium acetate was obtained from Sigma‐Aldrich/Merck and dissolved to 200 mM in Millipore water. Concanavalin A (Thermo Scientific) was dissolved to 30 µM in 200 mM ammonium acetate, and bovine serum albumin (BSA; Sigma‐Aldrich/Merck) was prepared between 5 nM and 10 µM in 200 mM ammonium acetate. Wild type (WT) NsXeR coding DNA in pET15b vector with His‐Tag and TEV cleavage site was used. The DNA for the F50Y and R100K mutant were constructed based on the WT using site‐directed mutagenesis. The mutations were confirmed by Microsynth Seqlab GmbH. WT, F50Y and R100K were transformed in *Escherichia coli* C43(DE3) strain. For expression, a preculture was grown overnight in LB medium with added 100 µg/mL ampicillin, at 37 °C and 220 rpm. Preculture cells were pelleted and washed twice in M9 medium. For main culture, the pelleted cells were transferred into M9 rich medium, containing all proteinogenic amino acids in addition (dilution: 1:100 WT and 1:50 mutants). The cell culture was grown at 37 °C and 220 rpm until OD_600 of 0.9 was reached. To induce protein expression, 1 mM isopropyl‐β‐D‐1‐thiogalactopyranoside (IPTG), 14 µM all‐trans retinal and 250 mg NaCl/500 mL were added. After three hours at 37 °C and 220 rpm, the cells were harvested and passed through a cell disruptor, three times at 1.85 kbar, followed by centrifugation at 158.420 rpm (43.000 rpm, rotor 45Ti) for 75 minutes at 4! °C, to pellet the membrane. The membrane pellet was solubilized overnight in buffer containing 1.5% (w/v) dodecyl‐β‐D‐maltoside (DDM) as detergent. The solubilized protein was purified by Ni‐NTA affinity chromatography and buffer exchanged to a imidazole free buffer via PD10 column. Immediately prior to analysis, Xenorhodopsin samples (≈50 µM) were desalted using Zeba Micro Spin Desalting Columns (Thermo Scientific, 7 kDa molecular weight cutoff) following the manufacturer's protocol, using a solution containing 20 mM Tris and 0.01% DDM.

### 4.2 Laser‐Induced Liquid Bead Ion Desorption Mass Spectrometry Instrumentation

LILBID‐MS spectra were acquired at room temperature on a home‐built LILBID ion source coupled to a time‐of‐flight (TOF) mass analyzer, as described previously by Morgner et al. [4]. In this setup, aqueous microdroplets of 70 µM diameter were generated at 10 Hz from a piezo‐driven droplet generator (Microdrop Technologies). Droplets were formed at ≈100 mbar and transferred into high vacuum ($\text{≈}10^{-5}$ mbar) where they were irradiated by infrared (IR) lasers. The subsequently desorbed ions were accelerated 15 µs after the IR irradiation and analyzed by a TOF analyzer.

To investigate the effect of delivering IR energy in two controllable pulses, we modified the standard LILBID source to accommodate two synchronized Nd:YAG lasers (Continuum Precision – *L*_1_, Innolas SpitLite 400 – *L*_2_). Both beams were tuned to 2.8 µm using a ${\mathrm{LiNbO}}_{3}$‐based optical parametric oscillator with ≈6 ns pulse durations and operated at 10 Hz to match droplet production. Per‐pulse energies were monitored with an optical power meter (Thorlabs, PM400) and pyroelectric energy sensors (Thorlabs, ES220C).

A pulse generator (Quantum Composer, 9520) timed the droplet ejection, the timings of *L*_1_ and *L*_2_ and the extraction pulse. The LD between *L*_1_ and *L*_2_ was scanned from −200 ns to + 200 ns (negative values: *L*_2_ first).

### 4.3 Data Processing

TOF Spectra were digitized with a PCI Express x4 Digitizer card (Spectrum Instrumentation) using their Labview drivers. The mean of 300 TOF spectra was used to calculate LILBID mass spectra. To determine peak integrals, a python script was used that integrates the peak area in a given peak range after doing a linear background subtraction.

## Funding

This study was supported by Deutsche Forschungsgemeinschaft (426191805 and 450648163).

## Conflict of Interest

The authors declare no conflicts of interest.

## Data Availability

The data that support the findings of this study are available on request from the corresponding author. The data are not publicly available due to privacy or ethical restrictions.
